# How is organisational fit addressed in Australian entry level midwifery job advertisements

**DOI:** 10.1186/s12913-021-06169-x

**Published:** 2021-02-27

**Authors:** Dianne Bloxsome, Courtney Glass, Sara Bayes

**Affiliations:** School of Nursing and Midwifery, Edith Cowan University, 270 Joondalup Drive, Joondalup, Western Australia 6027

**Keywords:** Employment, Midwifery, Organisational fit

## Abstract

**Background:**

Midwifery job retention is an ongoing global issue. Prior research has recognised that considering an individual’s attributes in relation to their work environment may assist in improving job satisfaction among midwives, leading to improved long-term job retention in the midwifery profession. The aim of this study was to evaluate whether, and how organisational fit is addressed in current entry level midwifery job advertisements within Australia.

**Methods:**

Midwifery jobs were searched for within 12 search engines, using the search term ‘midwife’, including Seek.com, Indeed.com, government employment websites for all Australian states and territories, and private health organisation websites. Data were extracted from eligible job advertisements by three independent researchers. Extracted data encompassed elements addressing person-job fit and person-organisation fit. Content analysis involving chi-square and Fischer exact tests were completed on extracted data.

**Results:**

Key findings demonstrate private health care organisations (29.2%) are more likely than public health care organisations (8.8%) to ask potential candidates to have additional qualifications, however, public health care organisations (34.1% vs. 16.7%) are more likely to ask for dual registration as a midwife and nurse. This is further supported by private health care organisations being more likely to refer to the candidate as a midwife (72.9% vs. 48.4%) than as a nurse. Private health care organisations more often noted access to support for employees and were more likely to mention access to employee assistance programs (41.7% vs. 13.2%), orientations (16.7% vs. 0%) and included benefits (72.9% vs. 42.9%). Clinical skills and personality traits were more frequently addressed in public health organisation advertisements; these included a requirement of employees to be accountable (49.5% vs. 6.3%), innovative (28.6% vs. 0%), have teamwork (69.2% vs. 52.1%) and conflict resolution skills (36.3% vs. 8.3%), and have knowledge of legislation (44.0% vs. 25.0%) and contemporary midwifery issues (28.6% vs. 4.2%).

**Conclusion:**

This study highlights that organisations employing midwives may be unwittingly contributing to the problem of midwife attrition through inattention to factors that endear midwives to workplaces in job advertisements. Further work developing employee selection and recruitment processes that are informed by the concept of person-job-organisation fit, is necessary.

## Background

There is considerable evidence that inability to retain midwives in the profession is an ongoing global issue [1]. Prior research has recognised that addressing an individual’s attributes in relation to their work environment may assist in improving job satisfaction among midwives, leading to improved long-term job retention in the midwifery profession [2]. One such recommendation to assist in this area is the implementation of the concept of fit [3]. ‘Fit’ has been described as the consideration of individuals’ attributes in relation to those of their work environment and has been explored in two ways: ‘person-job’ fit, which is an employee’s perception of comfort and compatibility within the culture of the organisation, and ‘person-organisation’ fit, which is the match between the characteristics of the potential employee to the job and culture of the organisation [4]. When all ‘fits’ (person-job-organisation) are in place, loyalty and greater effort on the part of the employee ensues and this in turn increases job satisfaction and the likelihood of employee retention [5]. Lam et al. [6] employed this concept in the hospitality sector and concluded that person-job fit and person-organisation fit improved work attitude and behaviour, improving employees’ performance and psychological wellbeing. The importance of getting the fit right is to ensure midwives remain both satisfied in their job and employed in the profession to improve retention. Midwives are the pillar to maternal and neonatal wellbeing and to ensure the longevity of the profession, consideration must be given to recruiting the right midwives to the right organisation [1]. The aim of this study was to evaluate whether, and how person-job-organisational fit is addressed in current entry level midwifery job advertisements within Australia.

## Methods

### Eligibility criteria

Australian entry level (classified by the entry level positions outlined in different organisational pay scales for registered caregivers, as either ‘Level One’ or ‘Grade Five’) midwifery jobs were included within this study. Graduate program positions and agency job advertisements were excluded. Midwife positions in a purely teaching and research environment (i.e. academia) were also excluded, as the focus of this study was to explore organisational fit within organisations hiring midwives for clinical duties. Any uncertainty in determining job advertisement eligibility for inclusion in the study were discussed and resolved by all authors.

### Search methods for included job advertisements

**Fig. 1:**
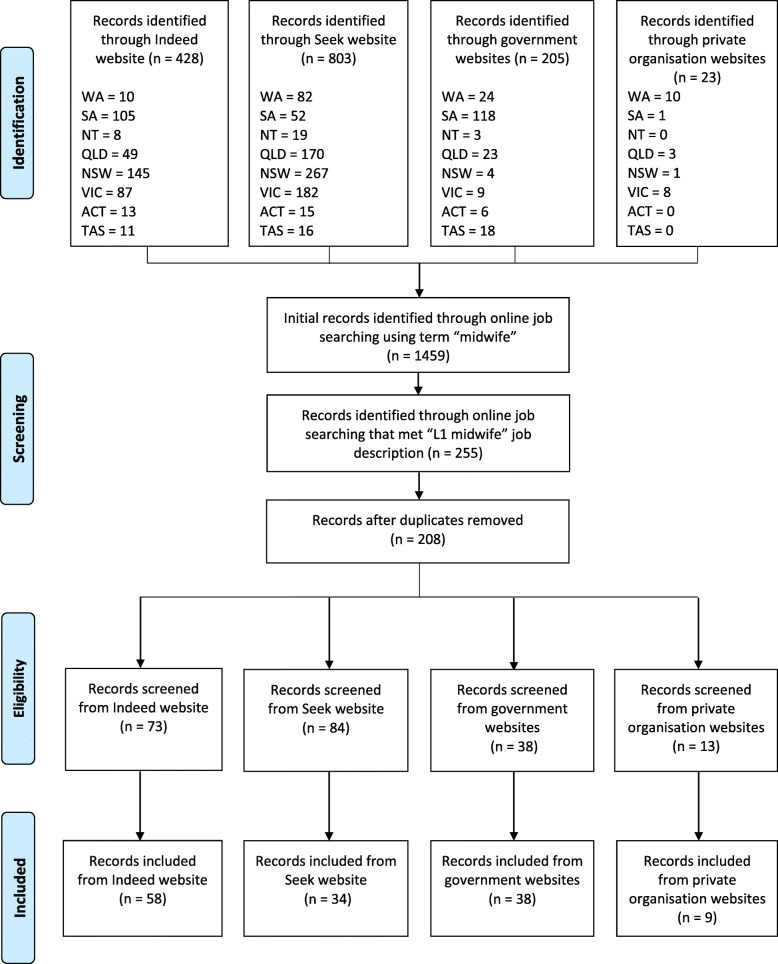
PRISMA flow diagram of job advertisement inclusion

National midwifery jobs were searched for within 12 search engines between January and February 2020 using the search term ‘midwife’. These included Seek.com, Indeed.com, private health care organisation website pages, and government employment pages for each state and territory in Australia. All job advertisements and job description forms that included ‘midwife’ within the job title were retrieved for analysis (Fig. 1). Once all job advertisements were obtained, they were screened by three independent research team members for duplicates and eligibility.

### Data extraction

Data were extracted from eligible job advertisements by all authors and collated into a spreadsheet within Microsoft Excel™ under either ‘person-job’ fit or ‘person-organisation’ fit [7]. Data extracted included details about the organisation including location (state/territory); whether the organisation offered primarily public or private health care (in Australia free healthcare is available to all citizens and permanent resident’s curtesy of the Federal Government. Private healthcare is a pay for service system that private health insurance companies can compensate individuals for services. Many Australians opt for private health care to choose when and who these services are provided by); job level or grade of the advertised position; organisational priorities (values, missions, visions, strategies); organisational structure; health care services available; facility details; resources available; model of care (woman-centred vs person-centred); language used to describe the candidate and skills, and line manager details. Applicant attributes were also captured, including qualifications; registration with governing bodies; experience; knowledge of contemporary midwifery issues; understanding of legislation, policy, and clinical governance; commitment to quality improvement, risk management and professional development; candidate skills (e.g. communications skills) and candidate personality traits (e.g. compassionate).

### Data analysis and synthesis

Content analysis was completed as per White and Marsh’s [8] methodology. This involved separating data into ‘chunks’ where text describing similar words or meanings were clustered together. These were then analysed to see if patterns in codes were occurring, after which they were sorted to assist in addressing the research question. Two groups were formed: one about employee traits and the other about employer/organisation traits. Once all data were extracted and categorised, data were cleaned in Microsoft Excel™ [7]. Data were then imported into IBM SPSS Version 25 where descriptive statistics were completed, summarising results into frequencies and percentages to outline the proportion of job advertisements that addressed each criterion [9]. Chi-square tests of independence and Fischer’s exact tests were completed to determine the relationship between organisation type (i.e. public versus private health care) and employee and employer attributes listed in job advertisements. A statistical significance level of *p* < 0.05 was utilised. Further multivariate analysis using binominal logistic regression was not completed due to the small sample of jobs retrieved compared to the amount of variables statistically significant from univariate analysis, with Bujang et al. [10] outlining an events per criterion sample size of at least 10–20 being required to reduce error. Findings from the study have been narratively synthesised and displayed within tables where appropriate.

## Results

The initial search of relevant jobs using the search term ‘midwife’ retrieved 1459 job advertisements (Fig. 1). After screening the advertisements to distinguish Level One or Grade Five midwifery job roles, a total of 255 job advertisements were identified. Two-hundred and eight advertisements remained after duplicates were removed. These records were screened for eligibility, with a total of 139 job advertisements meeting inclusion criteria for the study. The majority of eligible jobs were found through Indeed.com (*n* = 58), with government job websites (*n* = 38) and Seek.com (*n* = 34) providing a substantial offering of job advertisements. Only nine eligible jobs included in the study came from private health care organisation websites. A total of 91 job advertisements were classified as public health care roles and 48 as private health care.

### Employee qualifications and skill requirements

Table 1 highlights employee qualification and skill requirements that were addressed within the included job advertisements, and the proportion of job advertisements that addressed each category within public health care organisations compared to private health care organisations. Types of attributes identified in job advertisements of employees included obtained qualifications; registration with governing bodies; vehicle licence; experience; clinical skills; group facilitation skills, including education skills; whether the employee embraces patient or women-centre care; research skills; interpersonal and communication skills; teamwork and autonomy skills; time management skills; leadership skills; negotiation and delegation skills; decision-making and problem solving skills; conflict resolution ability; experience with computers; knowledge of contemporary nursing or midwifery issues; understanding of clinical governance and legislation; commitment to professional development; cultural awareness and shift flexibility.

**Table 1:** Employee qualification and skill requirements addressed in national job advertisements

Employee qualification/skill	Organisation type N (%)		***p***-value ^**c**^
	Public healthcare (***n*** = 91)	Private healthcare (***n*** = 48)	
Midwife qualification	25 (27.5)	17 (35.4)	.332
Nurse qualification	13 (14.3)	4 (8.3)	.308
Additional qualification(s)	8 (8.8)	14 (29.2)	***.002***
Registration with AHPRA	86 (94.5)	46 (95.8)	1.000 ^d^
Dual registration ^a^	31 (34.1)	8 (16.7)	***.030***
Vehicle licence	20 (22.0)	5 (10.4)	.092
Experience	60 (65.9)	36 (75.0)	.272
Clinical skills	64 (70.3)	36 (75.0)	.560
Group facilitations skills ^b^	25 (27.5)	3 (6.3)	***.003***
Embraces patient centred care	35 (38.5)	12 (25.0)	.111
Embraces woman centred care	37 (40.7)	18 (37.5)	.717
Research skills	24 (26.4)	3 (6.3)	***.004***
Communication skills	65 (71.4)	33 (68.8)	.742
Interpersonal skills	16 (17.6)	18 (37.5)	***.009***
Teamwork skills	63 (69.2)	25 (52.1)	***.046***
Autonomous	28 (30.8)	8 (16.7)	.071
Time management skills	24 (26.4)	14 (29.2)	.725
Leadership skills	37 (40.7)	10 (20.8)	***.019***
Decision making skills	7 (7.7)	2 (4.2)	.719 ^d^
Problem solving skills	30 (33.0)	10 (20.8)	.133
Delegation skills	9 (9.9)	2 (4.2)	.330 ^d^
Negotiation skills	32 (35.2)	4 (8.3)	***.001***
Conflict resolution skills	33 (36.3)	4 (8.3)	***< 0.001***
Computer skills	30 (33.0)	6 (12.5)	***.009***
Knowledge of contemporary midwifery issues	26 (28.6)	2 (4.2)	***.001***
Knowledge of contemporary nursing issues	21 (23.1)	1 (2.1)	***.001***
Understanding of clinical governance/legislation	40 (44.0)	12 (25.0)	***.028***
Commitment to quality improvement/risk management	40 (44.0)	15 (31.3)	.145
Commitment to professional development	55 (60.4)	31 (64.6)	.632
Cultural awareness	15 (16.5)	4 (8.3)	.184
Shift flexibility	53 (58.2)	29 (60.4)	.804

^a^ Dual registration as a nurse and midwife, ^b^ includes group education skills, ^c^ Chi-square test, ^d^ Fischer exact test, statistically significant (*p* < 0.05)

Chi-square tests of independence were performed to examine the relation between organisation type (i.e. public versus private health care) and employee qualifications and skills mentioned within job advertisements. A chi-square test of independence was completed to observe the relation between organisation type and additional qualifications obtained by the potential employee, which included additional postgraduate degrees, lactation support and additional clinical skills. The relation between these variables was significant, *X*^*2*^ (1, *N* = 139) = 9.79, *p* = .002, indicating private health care organisations (29.2%) were more likely than public health care organisations (8.8%) to request additional qualifications from potential employees in advertised jobs. Alternatively, a chi-square test of independence performed to examine the relation between organisation type and dual registration as a nurse and midwife dictated within the job advertisement showed a significant relationship between variables, *X*^*2*^ (1, *N* = 139) = 4.71, *p* = .03, suggesting public health care organisations (34.1%) were more likely compared to private health care organisations (16.7%) to request dual registration in job advertisements.

A chi-square test of independence was also completed to assess the relation between organisation type and knowledge of contemporary midwifery or nursing issues being addressed within the job advertisement. The relationship between organisation type and knowledge of contemporary midwifery issues was significant, *X*^*2*^ (1, *N* = 139) = 11.64, *p* = .001. Similarly, the relationship between organisation type and knowledge of contemporary nursing issues was also significant, *X*^*2*^ (1, *N* = 139) = 10.40, *p =* .001. These results indicate that public health care organisations (28.6% midwifery issues; 23.1% nursing issues) were more likely than private health care organisations (4.2% midwifery issues; 2.1% nursing issues) to include the requirement for potential employees to have sufficient knowledge of both contemporary midwifery and nursing issues. Public health care organisations (44.0%) were also more likely than private health care organisations (25.0%) to request potential employees to have adequate understanding of clinical governance and legislation, with the chi-square test of independence highlighting the significant relationship between variables, *X*^*2*^ (1, *N* = 139) = 4.82, *p* = .03.

Separate chi-square tests of independence were completed to examine the relation between organisation type and group facilitation/education skills, research skills, interpersonal skills, teamwork skills, leadership skills, negotiation skills, conflict resolution skills and computer literacy. The relationship between organisation type and each separate variable were significant. Public health care organisations were more likely than private health care organisations to request potential employees have group facilitation/education skills (*X*^*2*^ (1, *N* = 139) = 8.80, *p* = .003), research skills (*X*^*2*^ (1, *N* = 139) = 8.13, *p* = .004), teamwork skills (*X*^*2*^ (1, *N* = 139) = 3.98, *p* = .046), leadership skills (*X*^*2*^ (1, *N* = 139) = 5.52, *p* = .02), negotiation skills (*X*^*2*^ (1, *N* = 139) = 11.79, *p* = .001), conflict resolution skills (*X*^*2*^ (1, *N* = 139) = 12.55, *p* < .001) and computer skills (*X*^*2*^ (1, *N* = 139) = 6.86, *p* = .009). Alternatively, private health care organisations (37.5%) were more likely than public health care organisations (17.6%) to address interpersonal skills within job advertisements, *X*^*2*^ (1, *N* = 139) = 6.75, *p* = .009. The remaining skills analysed, including midwifery qualification, nursing qualification, registrations with AHPRA, vehicle licence, experience, clinical skills, patient centred care, woman centred care, communication skills, autonomy, time management skills, decision making skills, problem solving skills, delegation skills, commitment to quality improvement and risk management, cultural awareness and shift flexibility, did not show significant differences between organisation type addressing each element within job advertisements.

### Employee personality traits

Table 2 demonstrated the employee personality traits addressed in national midwifery job advertisements and the frequency the attributes were included in advertisements by public and private health care organisations. Separate chi-square tests of independence were completed to analyse the relation between organisation type and personality traits being addressed in job advertisements. Public health care organisations were significantly more likely than private health care organisations to highlight employee personality traits of accountability (*X*^*2*^ (1, *N* = 139) = 25.94, *p* < .001) and innovation (*X*^*2*^ (1, *N* = 139) = 16.87, *p* < .001). Private health care organisations (35.4%) were more likely than public health care organisations (8.8%) to request passionate future employees in job advertisements, *X*^*2*^ (1, *N* = 139) = 15.10, *p* < .001. Fischer’s exact tests were performed for variables that had less than five cases to assess the relationship between organisation type and personality traits addressed. Results demonstrated that private health care organisations were more likely than public health care organisations to address employee personality traits of motivation (*p* = .02, 2-tail) and caring nature (*p* = .01, 2-tail) in job advertisements. Alternatively, public health care organisations were more likely than private health care organisations to request potential employees have resilience (*p* = .002, 2-tail) and a calm nature (*p* = .03, 2-tail). Results from chi-square and Fischer exact tests of other personality traits including, compassionate; responsible; enthusiastic; intuitive; authentic; professional; analytical; self-aware; integrity; cooperative; personable; adaptable; respectful; role model and embraces organisations priorities (i.e. values, visions, and missions), were not significantly different when assessed by organisation type.

**Table 2:** Employee personality traits addressed in national job advertisements

Employee personality trait	Organisation type N (%)		***p***-value ^**a**^
	Public healthcare (***n*** = 91)	Private healthcare (***n*** = 48)	
Accountable	45 (49.5)	3 (6.3)	***< 0.001***
Compassionate	6 (6.6)	4 (8.3)	.737 ^b^
Passionate	8 (8.8)	17 (35.4)	***< 0.001***
Responsible	14 (15.4)	3 (6.3)	.118
Enthusiastic	10 (11.0)	11 (22.9)	.062
Intuitive	3 (3.3)	0 (0.0)	0.551 ^b^
Authentic	2 (2.2)	2 (4.2)	0.608 ^b^
Professional	7 (7.7)	5 (10.4)	0.752 ^b^
Innovative	26 (28.6)	0 (0.0)	***< 0.001***
Motivated	2 (2.2)	6 (12.5)	***.020*** ^***b***^
Analytical	0 (0.0)	1 (2.1)	.345 ^b^
Self-aware	0 (0.0)	1 (2.1)	.345 ^b^
Caring	0 (0.0)	4 (8.3)	***.013*** ^***b***^
Integrity	6 (6.6)	2 (4.2)	.714 ^b^
Cooperative	4 (4.4)	1 (2.1)	.659 ^b^
Personable	6 (6.6)	3 (6.3)	1.000 ^b^
Adaptable	5 (5.5)	2 (4.2)	1.000 ^b^
Resilient	14 (15.4)	0 (0.0)	***.002*** ^***b***^
Role model	5 (5.5)	3 (6.3)	1.000 ^b^
Respectful	7 (7.7)	1 (2.1)	.262 ^b^
Calm	9 (9.9)	0 (0.0)	***.027*** ^***b***^
Embraces organisations priorities	9 (9.9)	9 (18.8)	.139

^a^ Chi-square test, ^b^ Fischer’s exact test, statistically significant (*p* < 0.05)

### Employer traits

Organisational traits addressed within national midwifery jobs across public and private health care sectors included, organisational priorities (which encompassed values, mission and vision); organisation structure; outline of job role responsibilities; details of health services, facilities and resources provided; woman centred approach to care; language used to refer to candidate (i.e. registered midwife); commitment to professional development; encouragement of Aboriginal and/or Torres Strait Islander people to apply; welcoming and supportive environment; challenging environment; access to employee assistance programs, relocation support, benefits and orientations; and identification of line manager title (Table 3). Separate chi-square tests for independence or Fischer exact tests were completed to assess the relation between organisation type and employer traits addressed in job advertisements. Private health care organisations were significantly more likely than public health care organisations to refer to potential employees as “registered midwife” or “midwife” (*X*^*2*^ (1, *N* = 139) = 7.73, *p* = .005), address access to employee assistance programs (*X*^*2*^ (1, *N* = 139) = 14.38, *p* < .001), highlight access to an orientation (*p* < .001, 2-tail), and report benefits included within the job role (i.e. entitlements), *X*^*2*^ (1, *N* = 139) = 11.41, *p* = .001. Public health care organisations were significantly more likely than private health care organisations to address organisational structure (*X*^*2*^ (1, *N* = 139) = 28.59, *p* < .001) and identify the reporting line manager, *X*^*2*^ (1, *N* = 139) = 3.89, *p* = .049. The relationships between organisation type and the remaining variables were not significant.

**Table 3:** Employer traits addressed in national midwifery job advertisements

Employer trait	Organisation type N (%)		***p***-value ^**a**^
	Public healthcare (***n*** = 91)	Private healthcare (***n*** = 48)	
Organisational priorities discussed	71 (78.0)	32 (66.7)	.146
Organisational structure discussed	39 (42.9)	0 (0.0)	***<.001***
Job role outlined	80 (87.9)	43 (89.6)	.769
Details of health services provided within organisation	72 (79.1)	41 (85.4)	.365
Details of facilities and resources available within organisation	29 (31.9)	20 (41.7)	.250
Candidate referred to as Midwife only	44 (48.4)	35 (72.9)	***.005***
Woman-centred approach	37 (40.7)	18 (37.5)	.717
Commitment to professional development	39 (42.9)	21 (43.8)	.920
Aboriginal and Torres Strait Islander people encouraged to apply	16 (17.6)	8 (16.7)	.892
Welcoming/supportive environment	26 (28.6)	17 (35.4)	.406
Challenging environment	3 (3.3)	4 (8.3)	.197
Employee assistance program	12 (13.2)	20 (41.7)	***<.001***
Orientation provided	0 (0.0)	8 (16.7)	***<.001*** ^***b***^
Relocation support	0 (0.0)	2 (4.2)	0.118 ^b^
Benefits	39 (42.9)	35 (72.9)	***.001***
Reporting role identified	36 (39.6)	11 (22.9)	***.049***
Role employee reports to			.487
Nurse only	20 (22.0)	7 (14.6)	
Midwife only	3 (3.3)	2 (4.2)	
Nurse Midwife	10 (11.0)	1 (2.1)	
Other	3 (3.3)	1 (2.1)	
Not reported	55 (60.4)	37 (77.1)	

^a^ Chi-square square, ^b^ Fischer’s exact test, statistically significant (*p* < 0.05)

## Discussion

The findings from this study highlight the visible difference in the job description advertisements between public and private healthcare organisations across Australia for entry level midwives. First, there were key differences in job advertisements in relation to applicant personality traits for public and private health care organisations. Gardner et al. [11] conveys the importance of the prospective employer communicating measures to assess a person’s fit between their own personality and the organisation’s missions and values, and encouraging the candidate to ‘self-select out’ before recruitment if the attributes of both do not match. Kristof-Brown et al. [3] had previously also proposed this measure of values and personality to determine the extent of ‘person-job’ fit and ‘person-organisation’ fit. It is evident in the results of our study, however, that organisations characterise ‘fit’ differently and hence the words used around personality traits differ between public and private health care organisations [11].

Second, public healthcare organisations appeared to address candidates’ skills and knowledge while private healthcare organisations tend to focus on staff benefits and describe the extensive support they provide to the new employee, such as access to fitness programs, employee assistance programs and salary packaging. The challenges of practising in a private health care institution as a midwife who values woman-centred care and professional autonomy is reported in a recent study by Bradfield et al. [12], who acknowledged that midwives often struggle with working in an environment of overarching medical dominance but also recognise that midwives enjoy the flexibility and family friendly rostering that these employers tend to offer. Bloxsome et al. [13] similarly identified drivers underlying midwifery workforce retention to include flexibility to enable work life balance, however, these authors also found that working in accordance with one’s midwifery philosophy as well as a number of other factors is vital to staying – and thriving - in the profession and in a job. Whether or not the focus by private health services on employee benefits in job advertisements for midwives is tacit recognition of this phenomenon is not known.

Third, several of the job advertisements for midwives analysed for this study made reference to ‘nurses’ and/or ‘nursing’ to describe the candidate and/or role duties, and in some a nurse was identified as a midwives’ line manager. In addition, applicants for midwife positions in some organisations were required to be dual registered as a ‘registered nurse’ in the position description statements. In relation to the concept of fit, language is represented in organisational culture and often can be what makes organisations unique [11]. If an organisation can use the correct language, they are likely to promote common workplace values [14]. In a Canadian investigation into factors that affect midwifery practice, Behruzi et al. [15] found it was impacted negatively by other professions because of their lack of interest in midwives as well as differences in philosophy and scope of practice among healthcare professionals. There is no evidence in this regard from Australia, however, these findings are likely to extrapolate this setting. Historically in Australia, midwifery was recognised as a sub-specialty of nursing with standardised professional requirements, and non-nurse midwives were registered as nurses but were only permitted to practice midwifery [16]. This inconsistency impacted on the ability to project workforce planning and shaped the direction of midwifery across Australia. After much work from stakeholders, consumers and policy makers, midwifery was recognised as an independent separate profession in national health law in 2009; and instituted by the Australian Health Practitioner Regulatory Agency in 2010 [17]. Midwives have worked extremely hard to overcome this lack of recognition of their identity and of their role and remit as completely different to those of nurses, however, over a decade since midwifery was acknowledged and conferred as a profession in its own right in law, recruiting organisations continue to refer to midwives as nurses and consider it acceptable that midwives are managed and led by nurses.

Person-job-organisational fit plays a major role in the recruitment of the right midwives to the right organisation. It has the ability to take into account organisational goals and values, as well as the persons characteristics, including personality, values and needs to ensure the correct fit within the organisation.

## Conclusion

There is, for a range of reasons and like elsewhere in the world, a midwifery workforce retention issue in Australia, and strategies to address the problem are urgently required. This study has highlighted that organisations that employ midwives may be unwittingly contributing to the problem through their inattention to the factors that endear midwives to workplaces in their job advertisements. There is already a rich body of knowledge surrounding the need to address the global midwifery shortage and one such way is to consider greater sensitivity to midwives’ priorities at the first point of engagement with potential employees and in relation to the concept of ‘fit’.

The phenomenon of person-job-organisational fit has not previously been reported in midwifery, and attention to it would benefit both midwives, who would profit both personally and professionally from an increase in job satisfaction, and employers, whose midwifery workforce retention would be optimised. Future research should focus on exploring experiences of midwives in relation to person-job-organisational fit. Further work to develop employee selection and recruitment processes that are informed by the concept of person-job-organisation fit, is necessary.

## Data Availability

The datasets used and/or analysed during the current study are available from the corresponding author on reasonable request.
